# Antimicrobial Activity of Pyrazinamide Coordination Frameworks Synthesized by Mechanochemistry

**DOI:** 10.3390/molecules26071904

**Published:** 2021-03-28

**Authors:** Sílvia Quaresma, Paula C. Alves, Patrícia Rijo, M. Teresa Duarte, Vânia André

**Affiliations:** 1 Centro de Química Estrutural, Instituto Superior Técnico, Universidade de Lisboa, Av. Rovisco Pais, 1049-001 Lisboa, Portugal; quaresma.silvia@gmail.com (S.Q.); paula.alves.marques@tecnico.ulisboa.pt (P.C.A.); 2 Associação do Instituto Superior Técnico para a Investigação e Desenvolvimento (IST-ID), Av. Rovisco Pais, 1049-003 Lisboa, Portugal; 3 Centro de Investigação em Biociências e Tecnologias da Saúde (CBIOS), Universidade Lusófona de Humanidades e Tecnologias, Campo Grande 376, 1749-024 Lisboa, Portugal; p1609@ulusofona.pt; 4 Research Institute for Medicines (iMed. ULisboa), Faculty of Pharmacy, Universidade de Lisboa (UL), Av. Prof. Gama Pinto, 1649-003 Lisboa, Portugal; 5 Departamento de Engenharia Química, Instituto Superior Técnico, Universidade de Lisboa, Av. Rovisco Pais, 1049-001 Lisboa, Portugal

**Keywords:** mechanochemistry, supramolecular chemistry, pyrazinamide, antibiotics, antibiotic coordination frameworks

## Abstract

The urge for the development of a more efficient antibiotic crystalline forms led us to the disclosure of new antibiotic coordination frameworks of pyrazinamide, a well-known drug used for the treatment of tuberculosis, with some of the novel compounds unravelling improved antimycobacterial activity. Mechanochemistry was the preferred synthetic technique to yield novel compounds, allowing the reproduction of a 1D zinc framework, the synthesis of a novel hydrogen bonding manganese framework, and three new compounds with silver. The structural characterization of the novel forms is presented along with stability studies. The increased antimicrobial activity of the new silver-based frameworks against *Escherichia coli*, *Staphylococcus aureus,* and *Mycobacterium smegmatis* is particularly relevant.

## 1. Introduction

**Figure 1:**
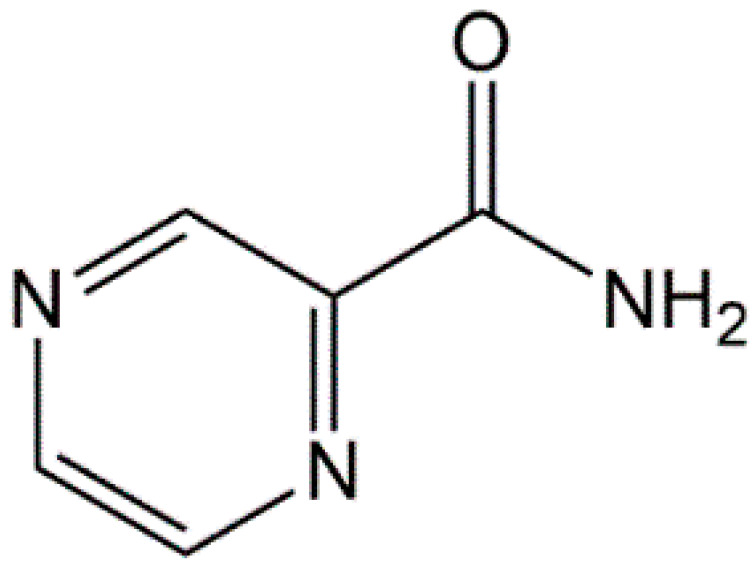
Pyrazinamide structure.

Pyrazinamide (Figure 1) is an antimycobacterial agent, with a well-established function as a front-line drug against tuberculosis that presents the most effective tuberculosis chemotherapy along with isoniazid, rifampicin, and ethambutol [1,2]. Indeed, the use of pyrazinamide in the treatment of tuberculosis [1] is recommend by the World Health Organization (WHO) [3] and it is in the WHO Model List of Essential Medicines [4].

Pyrazinamide plays a key role in shortening tuberculosis treatment from 9–12 months to 6 months. This capability seems to be correlated with its ability to kill a special bacterial population with low metabolic activity, which resides in acidic pH environments that are not killed by other tuberculosis drugs, and also its ability to kill persisting *Mycobacterium tuberculosis* during the initial intensive phase [1,2,5]. Despite its remarkable in vivo activity, pyrazinamide is not active against *M. tuberculosis* under usual culture conditions, at approximately neutral pH [1,5]. This discrepancy between the in vivo and in vitro activity of pyrazinamide reflects possible differences between in vivo and in vitro conditions that affect the drug activity. Even though it might not completely explain this discrepancy, it was found that pyrazinamide is active against *M. tuberculosis* only at acidic pH values [2,5].

A possible strategy to increase the efficiency of commercially available drugs is through the preparation of metal-organic frameworks (MOFs) for pharmaceutical purposes. MOFs are structures based on supramolecular chemistry principles [6,7] that can exhibit some of the highest porosities known, and, therefore, are suitable for diverse applications, including gas storage, luminescence, separation, catalysis, drug delivery [6,8,9,10,11,12,13], cancer therapy [14,15], and applications in imaging and sensing for therapeutic and diagnostic [12,16,17,18,19]. MOFs with biocompatible composition were reported to be viable drug carriers to overcome some of the problems related to drug delivery [9,20,21,22]. In particular, MOFs designed using antibiotics as ligands to yield antibiotic coordination frameworks (ACFs) were proposed to improve the antimicrobial activity [23,24,25], as well as the physicochemical properties of the antibiotics [26,27,28,29,30,31,32,33].

Accordingly, the development of new pyrazinamide coordination frameworks recurring to biocompatible metals envisaging synergistic effects can be explored as a viable pathway for the improvement of its antimycobacterial properties. A systematic analysis in the Cambridge Structural Database (CSD) [34] retrieved 48 pyrazinamide-based coordination compounds. Nevertheless, from this universe, the evaluation of the biological activity was studied only for a few compounds, which enclose Mn(II) and dicyanamide as the second ligand [35]; complexes of Cu(II), Fe(II), Co(II), Mn(II), and Ni(II) [36], and of Au(I) and Au(III) [37]. Some of these compounds were demonstrated to be more effective against *M. tuberculosis* than the free pyrazinamide, proving the potential of these systems for the improvement of the antimycobacterial properties of pyrazinamide. 

Even though MOFs and coordination complexes were traditionally obtained by solution techniques, including hydrothermal and solvothermal synthesis, there is a growing concern regarding the development of new and more sustainable synthetic alternatives. Mechanochemistry is one of these techniques that emerged in many areas, including in the synthesis of MOFs and metal complexes [38,39]. This is an environment-friendly technique that dismisses the use of solvent (neat grinding, NG) [40] or uses minimal amounts of solvent (liquid-assisted grinding, ion-, and liquid-assisted grinding, polymer-assisted grinding—LAG, ILAG, and POLAG, respectively) [41,42,43,44]. Besides the evident environmental benefits, this technique also demonstrated advantages concerning other important aspects, such as reaction time and efficiency. From the pharmaceutical perspective, it is important to mention that ball-milling, one of the possible mechanochemical approaches, is listed as a pharmaceutical particle technology to improve the solubility of poorly soluble drugs by reducing their particle size [28,39,45,46].

Herein, we disclose the mechanochemical synthesis and characterization of novel coordination and hydrogen bonding frameworks using pyrazinamide as a linker and biocompatible metals. In this study, besides *Escherichia coli* and *Staphylococcus aureus*, we used *Mycobacterium smegmatis*, a non-pathogenic model organism of *M. tuberculosis* [47], as a first approach to test new therapeutic compounds against tuberculosis [48]. The antibacterial activity assays revealed that, in some cases, these systems showed enhanced activity as compared to the free antibiotic.

## 2. Results and Discussion

**Scheme 1:**
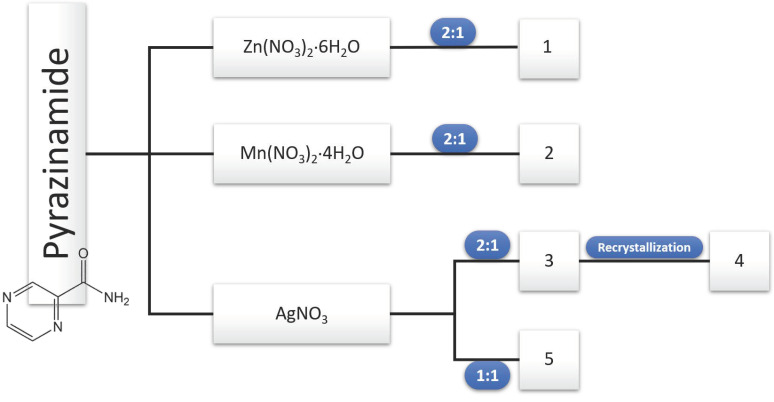
Summary of the neat grinding reaction conditions, depicting the stoichiometric ratios of pyrazinamide:metal salt used for the synthesis of compounds **1**-**3**, and **5**; **4** was obtained by the recrystallization of **3**.

Different biocompatible metallic ions (Zn(II), Mn(II) and Ag(I)) were tested in an attempt to synthesize coordination frameworks of pyrazinamide (PYR), recurring to mechanochemistry (Scheme 1). The herein called compound **1**, [Zn(PYR)_2_(H_2_O)](NO_3_)_2_, was already obtained from solution techniques (CSD refcode: HAYKUP) [34,49], but it was now reproduced using neat grinding. The discussion of its crystal structure is also included herein for comparison reasons. New compounds were attained with manganese ([Mn(PYR)_2_(H_2_O)_2_(NO_3_)](NO_3_), **2**) and silver nitrates ([Ag(PYR)_2_](NO_3_), **3**) and ([Ag(PYR)_2_(NO_3_)], **4**), using a (2:1) reaction stoichiometry. When using a (1:1) stoichiometric ratio with silver nitrate, a different novel compound, **5**, was obtained. Structural elucidation by powder and single-crystal X-ray diffraction data, as well as their physicochemical characterization, is presented and discussed, except for **4**, which is only structurally characterized, and **5** whose structural determination was precluded as no single crystals where it was possible to obtain. Antimicrobial assays were also performed to assess their antimicrobial activity, except for **4**.

### 2.1. Structural Characterization

**Figure 2:**
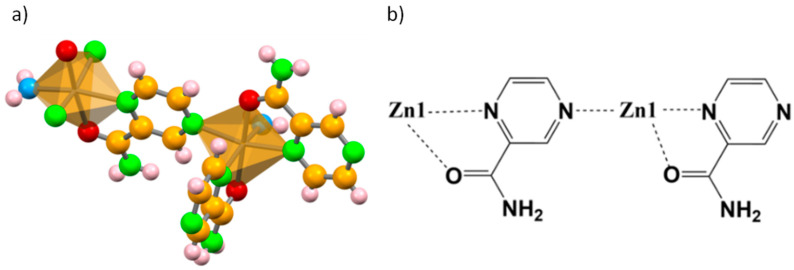
(**a**) View of the molecular structure of **1**, evidencing the coordination environment; (**b**) details on pyrazinamide coordination modes. Nitrate anions were omitted for clarity. Color code: C, yellow; O, red; Ow, cyan; N, green; H, white; and octahedral polyhedral representation, pale yellow.

The neat grinding reaction of pyrazinamide with zinc nitrate led to the formation of [Zn(PYR)_2_(H_2_O)](NO_3_)_2_, compound **1**. The comparison between the experimental PXRD pattern of **1** with the simulated pattern of HAYKUP (Figure S1) clearly confirmed the reproduction of the form reported previously [34,49]. The asymmetric unit of **1** contained one Zn(II) metal center, two pyrazinamide molecules, one water molecule, and two free nitrate anions. The Zn(II) metal center was six coordinated by two pyrazinamide ligands, both coordinating via the *N*,*O*-bidentate mode (distances ranging from 2.064(3) to 2.193(5) Å), but the second ligand further coordinated to a symmetry generated (x, 3/2 − y, −½ + z) metal center, with a distance of 2.179(5) Å. A water molecule fulfilled the octahedral geometry (2.042(6) Å). The equatorial plane was defined by N_4_N_5c_N_1_O_3W_ atoms, and the oxygen atoms O_1_ and O_2_ occupied the apical positions with an axial angle of 172.71(15) ° (O_1_–Zn_1_–O_2_) (Figure 2a,b and Table S1).

**Figure 3:**
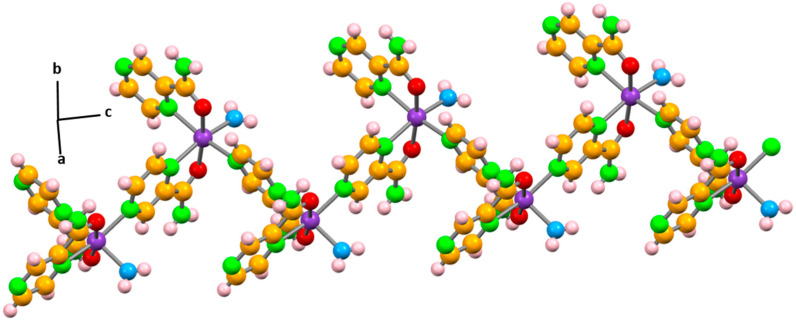
Representation of the 1D frameworks of **1**, extending along the c axis. Color code: Zn(II), purple C, yellow; O, red; Ow, cyan; N, green; and H, white.

One of the pyrazinamide ligands bridged the consecutive Zn(II) metal centers, promoting the formation of a 1D framework with zig-zag chains aligning along the c axis. The other crystallographically independent pyrazinamide coordinated to a single Zn(II) center, precluding the formation of 2D frameworks (Figure 3).

**Figure 4:**
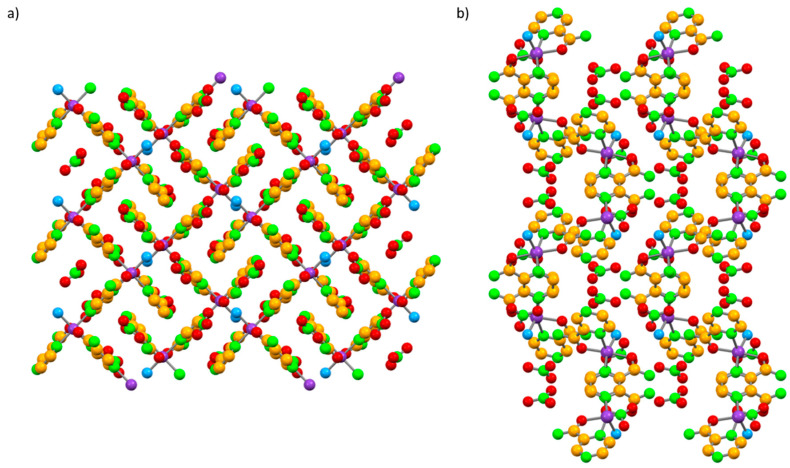
Supramolecular arrangement of **1** in a view along the (**a**) a axis and (**b**) the c axis. H atoms were omitted for clarity. Color code: Zn2+, purple; C, yellow; O, red; Ow, cyan; N, green; and H, white.

The overall supramolecular arrangement of **1** was based on hydrogen bond interactions established amongst the two free nitrate anions and the coordinated water molecule (Table 1). The water molecule interacted as a donor with both free nitrates via O_3W_–H_11W_⋯O_4_ (2.810(8) Å) and O_3W_–H_12W_⋯O_8_ (2.810(8) Å) hydrogen bonds, and as an acceptor from the amide NH_2_ fragment of pyrazinamide *N*,*O-*chelated through the N_3_–H_7_⋯O_3W_ (3.193(8) Å) hydrogen bond. This later amide NH_2_ fragment also established hydrogen bonds with the two free nitrates—N_3_–H_7_⋯O_5_ (3.163(8) Å) and N_3_–H_8_⋯O_7_ (2.937(8) Å). The crystal packing was further complemented by interactions between the amide NH_2_ fragment of the pyrazinamide that bridged the Zn(II) metal centers and a free nitrate anion (N_6_–H_9_⋯O_5_ (3.231(8) Å) and N_6_–H_10_⋯O_4_ (2.913(7) Å)), generating a 3D hydrogen bond framework (Figure 4a,b and Table 1).

**Figure 5:**
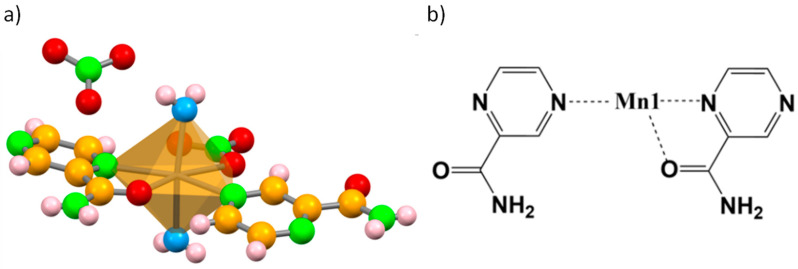
(**a**) View of the molecular structure of **2**. (**b**) Details on pyrazinamide coordination modes. Nitrate anions were omitted for clarity. Color code: C, yellow; O, red; Ow, cyan; N, green; H, white; and octahedral polyhedral representation, pale yellow.

Compound **2** was also prepared by the neat grinding pyrazinamide with manganese nitrate. The structure was determined from single crystal X-ray diffraction (SCXRD) data and formulated as [Mn(PYR)_2_(H_2_O)_2_(NO_3_)]NO_3_. PXRD data proved that the compound was obtained as a pure phase (Figure S2). The asymmetric unit of **2** was based on one Mn(II) metal center, two pyrazinamide molecules, two coordinated water molecules, one coordinate nitrate, and one free nitrate. Similarly to what was described for **1**, in compound **2**, the crystallographically independent pyrazinamide molecules showed different coordination behaviors, as one was chelated by the *N*,*O*-bidentate mode (distances of 2.3433(12) and 2.1570(1) Å, respectively), and the other only bound to the metal by the pyrazine-N atom (2.3508(12) Å). The Mn(II) coordination sphere was further completed by one nitrate (2.3161(13) Å) and two water molecules (2.1178(14) and 2.1640(13) Å). Mn(II) assumed a slightly compressed octahedral geometry, with the equatorial plane being defined by the O_1_N_4_O_3_N_1_ atoms, and the water molecules occupying the apical positions with an axial angle of 169.75(5)° (O_1W_–Mn_1_– O_2W_) (Figure 5a,b and Table S2).

**Figure 6:**
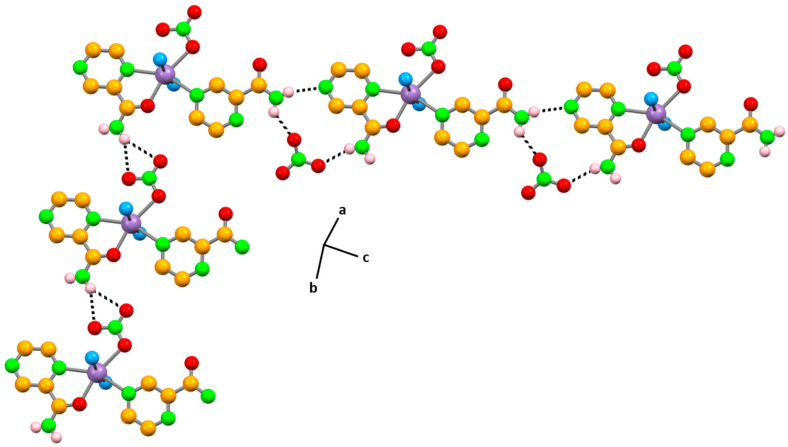
Representation of the hydrogen bonding frameworks of **2** extending along the b axis and the [101] direction, depicting the hydrogen bonds established in each layer. H atoms not involved in the hydrogen bonds were omitted for clarity. Color code: Mn(II), purple; C, yellow; O, red; Ow, cyan; N, green; and H, white.

Despite the different coordination behavior of the pyrazinamide molecules in **2**, there was no bridging of the consecutive metal centers, and therefore, this was a 0D structure. Its supramolecular arrangement could be described as hydrogen bonded chains extending both along the *b* axis and the [101] direction. The chains formed along b were attained through hydrogen bonds established between the NH_2_ group of the pyrazinamide molecule *N,O*-chelated, and the coordinated nitrate ion with the nitrogen atom N_3_ making bifurcated hydrogen bonds with both oxygen atoms of the coordinated nitrate: N_3_–H_2N_⋯O_4_ (3.368(2) Å), and N_3_–H_2N_⋯O_5_ (3.228(2) Å). The chains growing along [101] were based on N_6_–H_3N_⋯N_2_ (3.008(2) Å) hydrogen bonds established between the amide NH_2_ fragment of the *N,O*-chelated pyrazinamide and the pyrazine-N atom of consecutive crystallographically independent pyrazinamide molecules. This network was further reinforced by hydrogen bonds established between the NH_2_ group and the free nitrate, N_6_–H_4N_⋯O_7_ (2.880(2) Å) and N_3_–H_1N_⋯O_8_ (2.889(2) Å) (Figure 6, Table 1).

**Figure 7:**
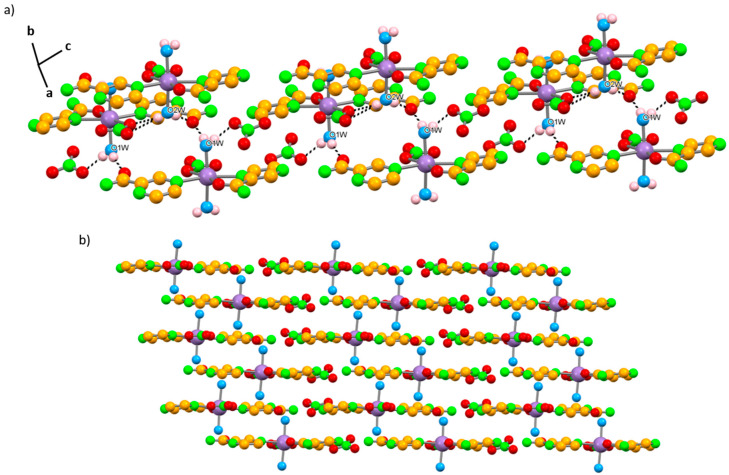
(**a**) Representation of parallel layers of **2** with the hydrogen bonds established between them. (**b**) Supramolecular arrangement of **2** in a view along b. For clarity, H atoms not involved in hydrogen bonds were omitted in (**a**); all H atoms were omitted in (**b**). Color code: Mn(II), purple; C, yellow; OPYR, red; Ow, cyan; N, green; H, white; and OH2O, blue.

Both water molecules interacted with the pyrazinamide amide O atom via O_1W_–H_1W_⋯O_2_ (2.7773(18) Å) and O_2W_–H_4W_⋯O_2_ (2.7278(17) Å). One of the water molecules further established hydrogen bonds with two oxygen atoms of the coordinated nitrate (O_2W_–H_3W_⋯O_3_ (2.9714(18) Å and O_2W_–H_3W_⋯O_4_ (3.186(2) Å), while the other water molecule interacted with the free nitrate anion (O_1W_–H_2W_⋯O_6_ (2.6723(19) Å) (Figure 7a). In the overall supramolecular arrangement, parallel layers of the complex aligned along the [101] plane, with the water molecules lying in between the layers (Figure 7b).

**Figure 8:**
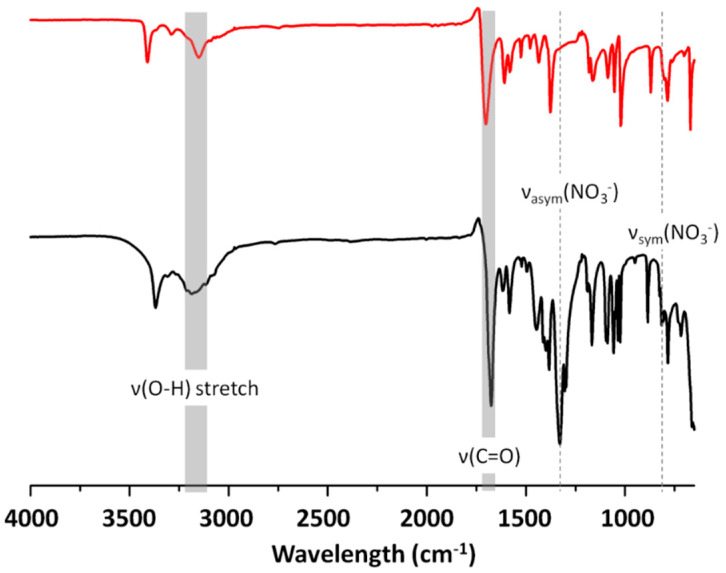
FTIR for pyrazinamide (**top**) compared with **2** (**bottom**) (y-axis: transmittance (%)).

Comparison of the FTIR spectra of pyrazinamide and **2** confirmed the presence of the coordinated water molecules, as the ν(O-H) stretch at ~3250 cm^−1^ was observed. The shift from 1704 to 1673 cm^−1^ of the carbonyl band confirmed that the carbonyl group participates in the coordination to Mn(II). In addition, the ν_asym_(NO_3_^−^) and ν_sym_(NO_3_^−^) stretching vibrations centered at ca. 1329 and 814 cm^−1^ ascertained the presence of the coordinated nitrate (Figure 8).

Using silver nitrate as the starting material, it was possible to attain three different reaction products—**3**, **4**, and **5**. Both **3** and **5** were obtained by neat grinding for 15 min—**3** was achieved starting from a (2:1) ratio, while **5** was achieved with a (1:1) ratio. In the recrystallization attempts to yield good quality single crystals of **3**, compound **4** was also identified. The structures of **3** and **4** were disclosed by SCXRD data and formulated as [Ag(PYR)_2_](NO_3_) and [Ag(PYR)_2_(NO_3_)], respectively. PXRD proved that compound **3** was prepared as a pure phase (Figure S3). Due to the low amount of crystals of **4** in the recrystallization bulk, only its structural characterization was presented herein. However, we could show **4** that was a novel compound through a comparison of the experimental PXRD patterns of **3** and **4** (Figure S4).

**Figure 9:**
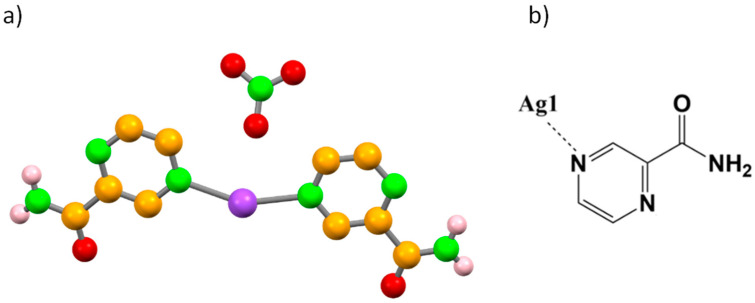
(**a**) View of the molecular structure of **3**. (**b**) Details on the pyrazinamide coordination mode. Color code: C, yellow; O, red; N, green; H, white; and tetrahedral polyhedral representation, violet.

The asymmetric unit of **3** was composed by a half Ag(I) metal center, one pyrazinamide molecule and half a non-coordinated nitrate. The Ag(I) metal center assumed a distorted linear geometry (N-Ag-N 154.1(3) °) coordinating to two pyrazinamide ligands by the pyrazine-N atom (2.230(5) Å) and giving rise to [Ag(PYR)_2_]^+^ ions (Figure 9a,b and Table S3).

**Figure 10:**
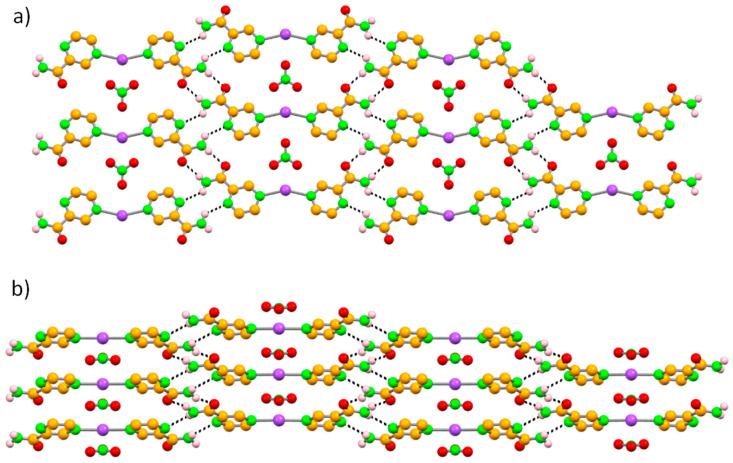
View of the molecular structure of **3**, in a view along the (**a**) a and (**b**) c axis. Color code: C, yellow; O, red; N, green; H, white; and Ag, violet.

This type of coordination led to a 0D structure, similarly to what was observed in **2**. The combination of $R_{2}^{2}\left(8\right)$ and $R_{2}^{2}\left(10\right)$ synthons generated a 2D hydrogen bonding framework that characterized the supramolecular arrangement of **3**. While the $R_{2}^{2}\left(8\right)$ homosynthons were established between neighboring amide moieties (2.883(8) Å), the $R_{2}^{2}\left(10\right)$ synthons arose from the interaction between the N-pyrazine and the NH_2_-amide groups (3.150(7) Å). Honeycomb-like layers of [Ag(PYR)_2_]^+^ ions were formed approximately within the [202] plane, with the nitrate counterions lying in the cavity of the honeycomb (Figure 10a). No hydrogen bonds were established with the nitrate ions, but each Ag(I) metal center was involved in short contacts with two nitrates in the same plane and two in neighboring planes (Figure 10b).

**Figure 11:**
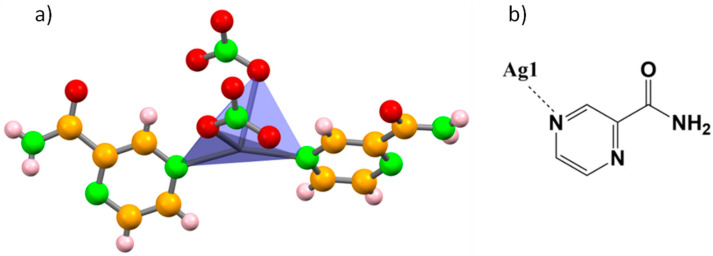
(**a**) View of the molecular structure of **4**, evidencing the coordination environment. (**b**) Details on the pyrazinamide coordination mode. Color code: C, yellow; O, red; N, green; H, white; and tetrahedral polyhedral representation, violet.

The asymmetric unit of **4** was formed by half Ag(I) metal center, one pyrazinamide molecule, and half a coordinated nitrate. The coordination of the nitrate precluded the linear geometry observed in **3**, and thus, in **4**, the Ag(I) metal center assumed a distorted tetrahedral geometry, coordinating to two pyrazinamide ligands by the pyrazine-N atom (2.227(4) Å) and two nitrates (2.227(4) Å) (Figure 11a,b, and Table S4). It is noteworthy that the coordination mode of pyrazinamide was maintained in **3** and **4**.

**Figure 12:**
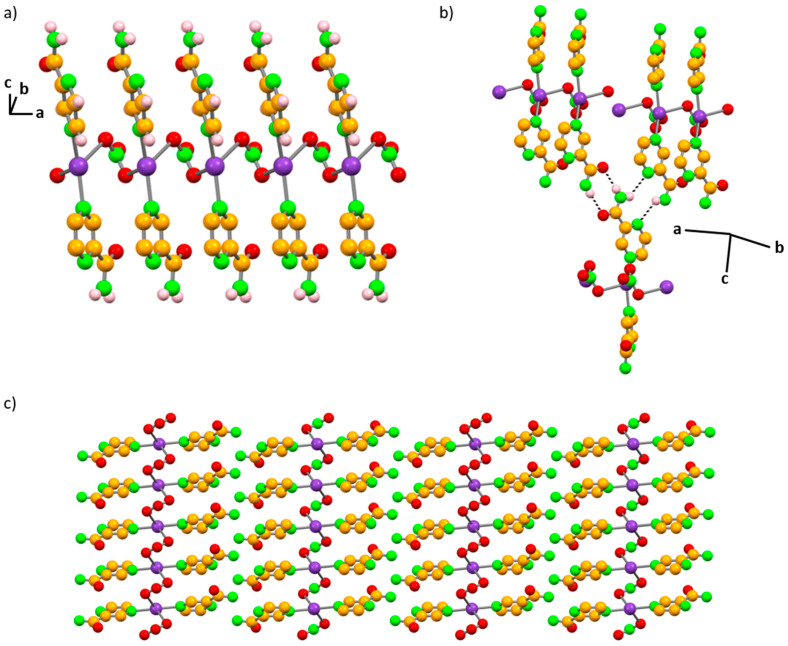
(**a**) Representation of one 1D chain of **4** running along a direction. (**b**) Highlight of the N_3_–H_1N_⋯N_2_ and N_3_–H_2N_⋯O_1_ hydrogen bonds interactions between the chains; (**c**) supramolecular arrangement of **4** in a view along b. For clarity, H atoms not involved in hydrogen bonds were omitted in (**a**) and (**b**); in (**c**) all H atoms were omitted. Color code: Ag(I) purple; C, yellow; O, red; N, green; and H, white.

In **4**, the nitrate ions connect to the consecutive Ag(I) centers, giving rise to a 1D framework that grows along *a* as chains (Figure 12a). Consecutive parallel chains pack in a head-to-head fashion and interact among them by N_3_–H_1N_⋯N_2_ (3.118(8) Å) and N_3_–H_2N_⋯O_1_ (2.883(8) Å) hydrogen bonds (Figure 12b,c and Table 1), giving rise to $R_{2}^{2}\left(10\right)$ and $R_{2}^{2}\left(8\right)$ synthons, respectively.

The formation of the novel compound **5** was confirmed by PXRD (Figure S5). Unfortunately, despite the many attempts carried out, its structural characterization was not possible.

**Figure 13:**
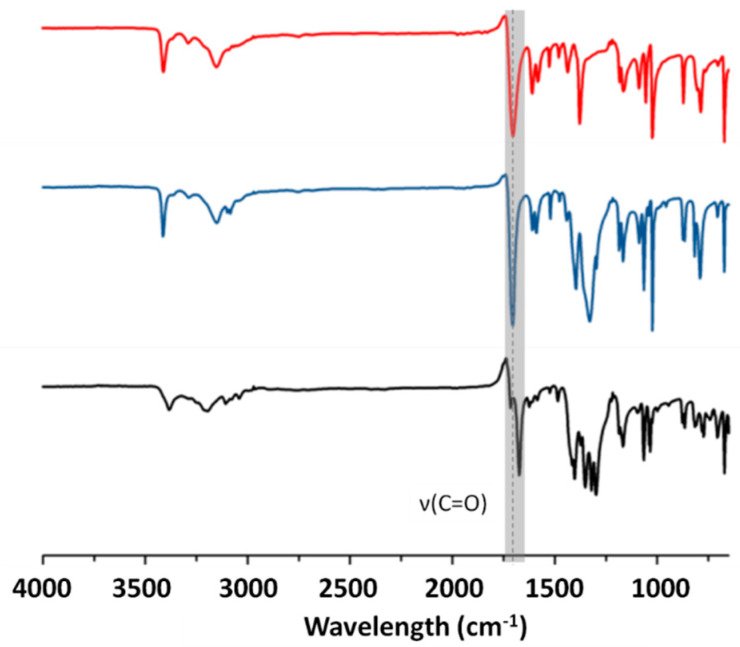
FTIR for pyrazinamide (top) compared with **3** (middle) and **5** (bottom) (y-axis: transmittance (%)).

Regarding the FTIR spectrum of **3**, it was possible to confirm that the carbonyl group did not participate in coordination to Ag(I), with its band being registered at 1704 cm^−1^ (Figure 13). On the other hand, a small peak was detected at 1704 cm^−1^ and a more intense peak appeared at 1674 cm^−1^ in the spectrum of **5** (Figure 13), and therefore this shifted peak might indicate that the coordination via the carbonyl group was a possibility in **5**, as seen in **1** and **2**.

**Figure 14:**
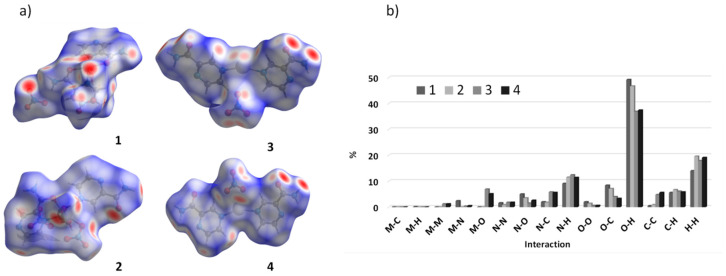
Hirshfeld surfaces for **1**–**4** (**a**), and graphical representation of the percentage of the various intermolecular contacts contributing to the Hirshfeld surfaces for **1**–**4** (**b**). Reciprocal contacts are included.

Hirshfeld surface analysis was used to further understand the intermolecular interactions in compounds **1**–**4** (Figure 14a and Figures S6–S10). The most representative interactions were indeed the O⋯H/H⋯O contacts with 49.2, 46.7, 36.9%, and 37.4%, for **1**, **2**, **3**, and **4**, respectively, which were represented by the two characteristic wings of hydrogen bonds. The H⋯H intercontacts showed large surfaces in the three structures, corresponding to the second most abundant contacts (14, 19.3, 17.9, and 19.1% for **1**, **2**, **3**, and **4**, respectively). The N⋯H/H⋯N interactions corresponded to 9.1, 11.5, 12.4, and 11.4% of the total interactions in **1**, **2**, **3**, and **4**, respectively, and were characterized by two sharp spikes pointing towards the upper and lower left of the plots corresponding to the described hydrogen bonds involving the N atoms. Besides these major interactions, the C⋯C, C⋯H/H⋯C, O⋯C/C⋯O, and O⋯O interactions were also observed, representing values below 7%. Interactions with the metal source were only observed in **1** (M⋯N/N⋯M 2.4%), **3** (M⋯O/O⋯M 6.9% and M⋯M 1.2%), and **4** (M⋯O/O⋯M 5.1% and M⋯M 1.2%) (Figure 14b).

### 2.2. Shelf and Thermal Stability of Compounds 2, 3, and 5

The determination of the physicochemical properties of the new compounds was of most relevance if potential pharmaceutical applications were envisaged. The thermal robustness of compounds **2**, **3**, and **5** was assessed by differential scanning calorimetry and thermogravimetric analysis (DSC/TGA).

**Figure 15:**
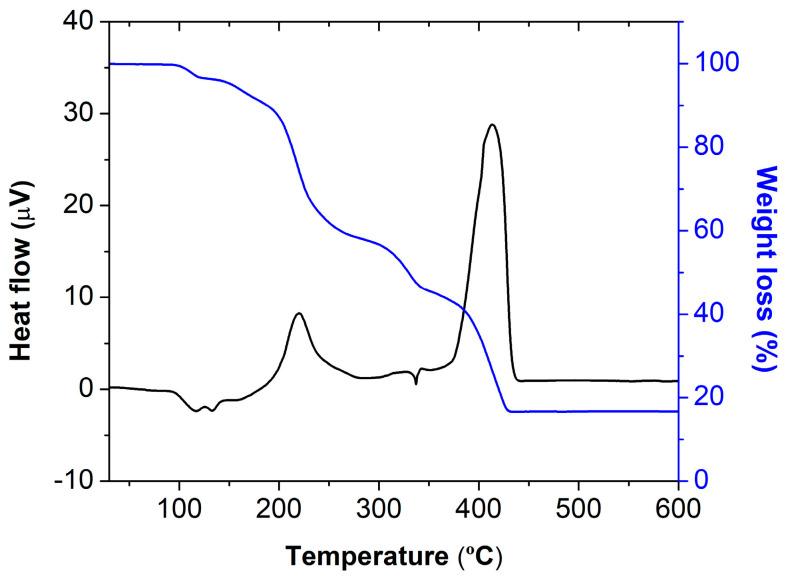
DSC (black) and TGA (blue) of **2**.

The TGA trace of **2** showed that it was thermally stable up to ca. 100 °C (Figure 15), temperature at which the release of the water molecules began, finishing at approximately 180 °C and corresponding to a total weight loss of 8.1%, (theoretical 7.8%). From ca. 180 °C onwards, an important and progressive weight loss of 49.2 % (theoretical 49.9%), associated with the complete oxidation and degradation of the organic fraction, was depicted. The final weight loss of 26.3% (theoretical 26.9%) at ca. 380 °C corresponded to the release of the nitrate ions to form MnO as a final product, as supported by the percentage of the residues, which was in accordance with the theoretical value of 15.4% (experimental 16.4%) for the remaining MnO.

**Figure 16:**
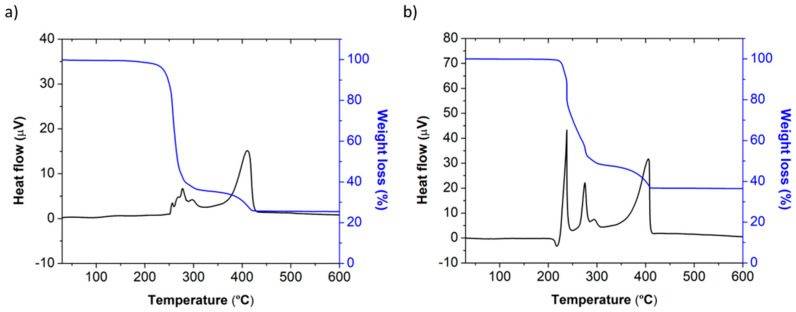
DSC (black) and TGA (blue) of (**a**) **3** and (**b**) **5**.

Regarding **3** and **5**, the TGA curves showed that they were both stable until ca. 210 °C, temperature at which the first weight loss was observed (Figure 16a,b, respectively). This assured the absence of water (either coordinated or of crystallization) in both compounds. For **5**, due to the uncertainty about the crystal structure, further conclusions are not established. In **3**, the weight loss of 58.2% (theoretical 57.3%) at ca. 170 °C was associated with the complete oxidation and degradation of the organic fraction, and the final weight loss of 15.2% (theoretical 14.9%) at ca. 280 °C corresponded to the release of the nitrate ion. These values were in accordance with the theoretical value of 27.8% (experimental 26.6%) for the remaining Ag_2_O.

Regarding the shelf stability of compounds **1**–**3**, we could conclude that these were stable on the shelf at room temperature for at least 14 months (Figure S11).

### 2.3. Antimicrobial Activity of the Compounds

The synthesized compounds **1**–**3** and **5** and the corresponding starting materials were tested against *Escherichia coli* (Gram-negative bacteria), *Staphylococcus aureus*, and *Mycobacterium smegmatis* (Gram-positive bacteria) to assess their antimicrobial activity. *M. smegmatis* was used as a non-pathogenic model organism of the *M. tuberculosis,* responsible for tuberculosis, which remained a leading cause of death worldwide, both in developing and developed countries. Considering the minimal inhibitory concentration (MIC) values found for each compound (Table 2), it was clear that the silver compounds were the most active, benefiting from the synergistic effects of Ag(I).

The preliminary results obtained against the assayed microorganisms showed moderate antimicrobial activity, with the coordination compounds **1**, **3**, and **5** showing a significant decrease in the MBC against *M. smegmatis*. Importantly, the Ag-based compounds **3**, and **5** showed a significant increase in the antibacterial activity against the three bacteria strains tested herein. This increase was more pronounced against *E. coli* and *M. smegmatis* than against *S. aureus.* A possible explanation for this might be the fact that *M. smegmatis* presents high similarities to *E. coli* regarding some cell biology parameters, such as cell volume, ribosome number, and ribosome density [50,51]. Interestingly, genome-based phylogeny studies suggest that mycobacteria are closely related to Gram-positive bacteria but also share properties with Gram-negative bacteria, like the non-retention of the Gram staining [52].

Looking specifically to the MIC values obtained for *M. smegmatis*, it is clear that coordination compounds **3** and **5** display antimycobacterial activity four to eight times higher than the free pyrazinamide, respectively.

These promising outcomes result from the synergistic effect between the antibiotic itself and Ag(I), a metal known for its own antimicrobial effects. The proximity between the minimum bactericidal concentrations and the minimum inhibitory concentration values for both **3** and **5** reveal their bactericidal effect against the three tested microorganisms. The obtained results emphasize the importance of this type of antibacterial coordination frameworks to augment the antibacterial activity of already commercially available antibiotics, especially to fight diseases with a huge socio-economic impact like tuberculosis.

## 3. Experimental Details

### 3.1. Reagents

The following reagents and solvents were purchased from commercial sources and used without further purification—pyrazinamide (C_5_H_5_N_3_O, 97.5 % Sigma-Aldrich, Munich, Germany), zinc nitrate hexahydrate (Zn(NO_3_)6H_2_O, 98.5–102%, Scharlau, Barcelona, Spain), manganese nitrate tetrahydrate (Mn(NO_3_)_2_.4H_2_O, 98%, Alfa Aesar, Germany), silver nitrate (AgNO_3_, 99%, Merck, Darmstadt, Germany), methanol (CH_3_OH, 99.9%, Carlo-Erba, Chaussée du Vexin, France), ethanol (C_2_H_5_OH, 99%, Carlo-Erba, Chaussée du Vexin, France), acetone (C_3_H_6_O, 99,8%, Merck, Darmstadt, Germany), and dimethyl sulfoxide (DMSO, 99%, Fluka, Buchs, Switzerland).

### 3.2. Synthesis of the Compounds

The reaction products **1**–**3** and **5** were synthesized via neat grinding using pyrazinamide and different metal salts (Mn(NO_3_)_2_4H_2_O, Zn(NO_3_)_2_6H_2_O and AgNO_3_) for the defined periods of time listed in Table 3. The reactions were carried out in a Retsch MM400 ball mill operating at 28 s^−1^, using two 7 mm stainless steel balls inside 15 mL stainless steel grinding jars, with a snap closure.

Compound **4** was obtained in the recrystallization of **3** in water, through slow evaporation of the solvent, at room temperature.

### 3.3. General Characterization

Powder X-Ray Diffraction (PXRD) data were collected in a D8 Advance Bruker AXS θ–2θ diffractometer (Bruker, Karlsruhe, Germany), equipped with a LYNXEYE-XE detector, copper radiation source (Cu Kα, λ = 1.5406 Å), operated at 40 kV and 30 mA, with the following data collection parameters—3–60° 2θ range, a step size of 0.02°, and 0.6 s per step and 3–37° 2θ range, step size of 0.02° and 0.5 s per step. The diffractograms were used to ascertain bulk material purity of the compounds **1**–**3**, by comparing the calculated (from SCXRD data) and experimental PXRD patterns, and to ascertain the formation of the new reaction product (**5**), in comparison with the starting materials. MERCURY 2020.2.0 [53] was used to obtain the diffraction patterns of the pyrazinamide polymorphs and of the calculated pattern obtained from single-crystal data.

Fourier Transform Infrared Spectroscopy (FTIR) measurements were recorded on a Thermo Nicolet 6700 spectrometer (Waltham, MA, USA) with attenuated total reflectance (ATR) mode by averaging 32 scans at a maximum resolution of 4 cm^−1^, registering the spectra at a wavelength interval of 4000–650 cm^−1^.

Combined Thermogravimetric Analysis–Differential Scanning Calorimetric (TGA–DSC) were carried out using a Setsys Evo 16 SETARAM TG-DTA 92 thermobalance (Caluire, France) under air flow, with a heating rate of 10 °C min^−1^. The samples weights were in the 8–20 mg range.

### 3.4. Single Crystal X-ray Diffraction Studies (SCXRD)

Suitable single crystals of **2**, **3**, and **4** were obtained by slow evaporation of the as-synthesized products from acetone, water, and methanol solution, respectively. Crystal structures of **2, 3**, and **4** were determined from SCXRD data. Data were collected on a Bruker AXS-KAPPA APEX II (Bruker, Karlsruhe, Germany), with graphite-monochromated radiation (Mo Kα, λ = 0.71073 Å) at 293 K. X-ray generator was operated at 50 kV and 30 mA and the APEX2 program was monitored for data collection. Data were corrected for Lorentzian polarization and absorption effects using SAINT [54] and SADABS [55] programs. SHELXT 2014/4 [56] was used for structure solution and SHELXL 2014/7 [57] was used for full matrix least-squares refinement on *F*^2^. These two programs were included in the WINGX-Version 2014.1 [58] program package. A full-matrix least-squares refinement was used for the non-hydrogen atoms with anisotropic thermal parameters. The H_CH_ were inserted in idealized positions and allowed to refine in the parent carbon atom. The hydrogen atoms of the NH_2_ group of **2** and **4** and of the water molecules of **2** were located from the electron density map and allowed to refine freely. Additionally, in **3**, the NH_2_ group was located from the electron density map but the N-H distances were restrained. MERCURY 2020.2.0 [53] was used for packing diagrams and for polyhedral representation. PLATON [59] was used for the determination of hydrogen bond interactions. Table 4 summarizes the data collection and refinement details. Crystallographic data of complexes **2**, **3**, and **4** were deposited at the Cambridge Crystallographic Data Centre (CCDC 2063304-2063306).

### 3.5. Antibacterial Activity Assays

Gram-negative (*Escherichia coli* (ATCC 25922) and Gram-positive bacteria (*Staphylococcus aureus* (ATCC 25923) and *Mycobacterium smegmatis* (ATCC 607)) were selected as model organisms for the determination of the minimum inhibitory and bactericidal concentrations (MIC and MBC, respectively) of the synthesized compounds and respective starting materials.

These values were determined by the microdilution method [60,61]. In brief, 100 μL of the Mueller-Hinton liquid culture medium were added to all 96-wells of the microplate, and 100 μL of the testing compounds at a concentration of 1 mg⋅mL^−1^ in DMSO were added to the first well. Serial dilutions of (1:2) were performed and 10 μL of bacterial inoculum was added to each well. Then, the microplates were incubated for 24 h and 48 h, at 37 °C. After 24 h or 48 h, the plates were visually analyzed and the lower concentration well, where no visible bacterial growth could be detected, was considered as the MIC value. Then, the content of the wells without active bacterial growth (within the range concentrations equal and higher to the MIC) was used as inoculum to be spread onto a new sterile Mueller-Hinton solid culture medium plates. These solid medium plates were incubated at 37 °C for 24 h, to allow MBC value determination, which was the lowest concentration with no visible bacterial growth.

## 4. Conclusions

Neat grinding successfully led to the formation of four novel compounds, **2**–**5**, and the reproduction of **1**. Even though **1**, a 1D framework with Zn(II), was previously disclosed [34,49], herein we proposed mechanochemistry as a sustainable alternative for the synthetic procedure. Compounds **2**–**5** were unveiled for the first time herein, but the full structural elucidation was only possible for **2**, **3**, and **4**, due to the impossibility of growing good quality single crystals of **5**. For **4**, only its structural characterization was possible due to the low amount of sample obtained.

With Zn(II) and Ag(I) nitrates, the (2:1) stoichiometry directly led to the formation of 1D coordination frameworks (**1** and **4**), while the final product resulting from the reaction with Mn(II) nitrate corresponded to a complex (**2**), as well as **3**, which results from the reaction with Ag(I) nitrate. Nevertheless, in **1**–**4**, hydrogen bonding frameworks were formed. Furthermore, it was noticed that, as a ligand, pyrazinamide was able to generate different coordination modes. With Zn(II) (**1**) and Mn(II) (**2**), it coordinated via both the *N*,*O*-amide moiety and the pyrazine-N atoms, while with Ag(I) (**3** and **4**) it only coordinated by the pyrazine-N atom.

Compounds **2**, **3**, and **5** were stable on shelf for at least 14 months and they were also stable at least until 100 °C. Compound **2** was thermally stable up to ca. 100 °C temperature, when the water molecules were released from the structure. Compounds **3** and **5** were stable until ca. 200 °C, confirming the absence of water molecules in **3** and also proving that absence in **5**.

These compounds were promising with regards to the improvement of pharmaceutical properties compared to the free antibiotic and these results were indeed encouraging to further study potential applications in the pharmaceutical field. Even though the MBC value obtained with **1**, in which Zn was used, already represented an asset, the best results were indeed obtained with the Ag-based compounds. The antimicrobial activity tests showed that the Ag-pyrazinamide compounds **3** and **5** displayed at least four times higher antibiotic activity than pyrazinamide against *E. coli* (a Gram-negative model bacteria), *S. aureus* (a Gram-positive model bacteria), and *M. smegmatis* (a Gram-positive non-pathogenic bacteria used as a model on the *Mycobacterium* genus works). The bactericidal effect obtained with these two compounds is an additional positive result that encourages further studies with similar compounds.

## Figures and Tables

**Table 1:** List of the main hydrogen bonds found for compounds **1**–**4**.

Compound	Symmetry Operation	D–H⋯A	*d*(D-H) (Å)	*d*(H⋯A) (Å)	*d*(D⋯A) (Å)	(DHA) (°)
**1**	*1 − x,1 − y, 1 − z*	N_3_–H_7_⋯O_3W_	0.86	2.50	3.193(8)	138
	*1 − x,1 − y, 1 − z*	N_3_–H_7_⋯O_5_	0.86	2.42	3.163(8)	144
	*x, y, z*	N_3_–H_8_⋯O_7_	0.86	2.08	2.937(8)	172
	*2 − x, 1 − y, 1 − z*	N_6_–H_9_⋯O_5_	0.86	2.41	3.231(8)	161
	*2 − x, ½ + y, ½ − z*	N_6_–H_10_⋯O_4_	0.86	2.07	2.913(7)	166
	*x, y, z*	O_3W_–H_11W_⋯O_4_	0.75(6)	2.06(7)	2.781(8)	162(9)
	*1 − x,1 − y, 1 − z*	O_3W_–H_12W_⋯O_8_	0.76(7)	2.05(7)	2.810(8)	177(7)
**2**	*1 − x, 2 − y, − z*	N_3_–H_1N_⋯O_8_	0.77(2)	2.15(3)	2.889(2)	159(2)
	*2 − x, 1 − y, 1 − z*	O_1w_–H_1w_⋯O_2_	0.86(3)	1.94(3)	2.7773(18)	166(2)
	*x, 1 + y, z*	N_3_–H_2N_⋯O_4_	0.89(3)	2.54(2)	3.368(2)	155(2)
	*x, 1 + y, z*	N_3_–H_2N_⋯O_5_	0.89(3)	2.41(3)	3.228(2)	153(2)
	*x, y, z*	O_1w_–H_2w_⋯O_6_	0.81(3)	1.87(3)	2.6723(19)	169(3)
	*1 + x, y, 1 + z*	N_6_–H_3N_⋯N_2_	0.81(3)	2.23(2)	3.008(2)	163(2)
	*1 − x, 1 − y, 1 − z*	O_2w_–H_3w_⋯O_3_	0.75(3)	2.28(3)	2.9714(18)	155(3)
	*1 − x, 1 − y, 1 − z*	O_2w_–H_3w_⋯O_4_	0.75(3)	2.51(3)	3.186(2)	150(3)
	*2 − x, 2 − y, 1 − z*	N_6_–H_4N_⋯O_7_	0.87(3)	2.20(3)	2.880(2)	135(2)
	*1 − x, 1 − y, 1 − z*	O_2w_–H_4w_⋯O_2_	0.89(3)	1.84(3)	2.7278(17)	178(3)
**3**	*x, y, z*	N_3_–H_1N_⋯N_2_	0.90(5)	2.44(8)	2.733(7)	100(6)
	*2 − x 1 − y, 1 − z*	N_3_–H_1N_⋯N_2_	0.90(5)	2.44(8)	3.150(7)	137(6)
	*1 − x, 1 − y, 2 − z*	N_3_–H_2N_⋯O_1_	0.90(6)	2.02(6)	2.888(7)	164(6)
**4**	*x, − y, 1 − z*	N_3_–H_1N_⋯N_2_	0.81(8)	2.48(8)	3.118(8)	136(7)
	*2 − x, 1 − y, 1 − z*	N_3_–H_2N_⋯O_1_	0.79(8)	2.10(8)	2.883(8)	177(8)

**Table 2:** Minimal inhibitory concentration (MIC) and minimal bactericidal concentration (MBC) of each reagent and synthesized compound for *Escherichia coli* (Gram-negative bacteria), *Staphylococcus aureus*, and *Mycobacterium smegmatis* (Gram-positive bacteria).

Compounds	*E. coli*		*S. aureus*		*M. smegmatis*	
	MIC * (µg/mL)	MBC * (µg/mL)	MIC ** (µg/mL)	MBC * (µg/mL)	MIC ** (µg/mL)	MBC * (µg/mL)
Pyrazinamide	>62.5	250	>250	500	>62.5	125
(1)	>62.5	500	>250	500	>62.5	62.5
Zn(NO_3_)_2_	>62.5	500	>250	500	>62.5	62.5
(2)	>62.5	500	>250	500	>62.5	125
Mn(NO_3_)_2_	>62.5	500	>250	500	>62.5	125
(3)	15.63	31.25	62.5	125	15.63	31.25
(5)	7.81	31.25	62.5	125	7.81	31.25
AgNO_3_	7.81	31.25	62.5	125	7.81	7.81
Positive control	<0.49 (NOR)	Nd	0.98 (VAN)	Nd	<0.49 (VAN)	Nd
Negative control (DMSO)	62.5	Nd	250	Nd	62.5	Nd

Legend: NOR—norfloxacin; VAN—vancomycin; Nd—Not determined; * after 24h; ** after 48h. Note: The antimicrobial effect activity of pyrazinamide, **1**, **2**, and the corresponding metal salts are limited by the effect of DMSO.

**Table 3:** Neat grinding experimental conditions for the synthesis of compounds **1–3** and **5.**

Pyrazinamide (PYR)	Metal Salt (M)	Molar Ratio (PYR:M)	Time	Resulting Compound
0.0907 g (0.74 mmol)	Zn(NO_3_)_2_⋅6H_2_O 0.1105 g (0.37 mmol)	2:1	10 min	(1)
0.0992 g (0.81 mmol)	Mn(NO_3_)_2_⋅4H_2_O 0.1036 g (0.41 mmol)	2:1	15 min	(2)
0.1183 g (0.96 mmol)	AgNO_3_ 0.0829 g (0.49 mmol)	2:1		(3)
0.0840 g (0.68 mmol)	AgNO_3_ 0.1171 g (0.69 mmol)	1:1		(5)

**Table 4:** Crystallographic data for **2**–**4**.

	2	3	4
Chemical formula	C_10_H_10_N_6_O_2_Mn⋅2H_2_O⋅2NO_3_	C_10_H_10_N_6_O_2_Ag⋅NO_3_	C_10_H_10_N_6_O_2_Ag⋅NO_3_
Formula weight	461.23	416.12	416.12
Temperature (K)	293	293	293
Wavelength (Å)	0.71073	0.71073	0.71073
Crystal form, color	Block, colorless	Block, colorless	Plate, colorless
Crystal size (mm)	0.17 × 0.08 × 0.03	0.14 × 0.10 × 0.10	0.12 × 0.12 × 0.10
Crystal system	Triclinic	Monoclinic	Monoclinic
Space group	*P* $\bar{1}$	*P*2_1_/*m*	*P*2/*c*
*a* (Å)	6.9866(3)	3.6132(11)	3.5903(4)
*b* (Å)	9.2273(4)	27.735(8)	6.7181(6)
*c* (Å)	14.1882(5)	6.6273(19)	27.610(3)
*α* (°)	90.584(3)	90.00	90.00
*β* (°)	99.285(2)	94.007(9)	91.149(5)
*γ* (°)	101.460(2)	90.00	90.00
*V* (Å^3^)	883.85(6)	662.5(3)	665.81(11)
Z	2	2	2
*d* (mg.m^−3^)	1.733	2.086	2.076
*µ* (mm^−1^)	0.820	1.564	1.556
*F(000)*	470	412	412
*θ* range (°)	2.630–30.976	3.081–26.510	2.952–26.563
Reflections collected/unique	15432/5513	4443/1381	5186/1374
R_int_	0.0349	0.0470	0.0301
GOF	1.020	1.132	1.217
Final R indices [*I* > 2*σ*(*I*)]	R_1_ = 0.0357, wR_2_ = 0.0892	R_1_ = 0.0630, wR_2_ = 0.1316	R_1_ = 0.0577, wR_2_ = 0.1256
Indices all data	R_1_ = 0.0476, wR_2_ = 0.0938	R_1_ = 0.0780, wR_2_ = 0.1372	R_1_ = 0.0620, wR_2_ = 0.1273

## Data Availability

Crystallographic data of compounds **2**–**4** were deposited at the Cambridge Crystallographic Data Centre (CCDC 2063304-2063306).

## Supplementary Materials

The following are available online. Figure S1. PXRD patterns of the experimental (top) (1) and simulated (bottom) of HAYKUP; Figure S2. PXRD patterns of the experimental (top) and simulated (bottom) 2; Figure S3. PXRD patterns of the experimental (top) and simulated (bottom) 3; Figure S4. PXRD patterns of the simulated crystal of 4 (top) compared with the experimental 3 (bottom); Figure S5. PXRD patterns of the experimental of 5 (red) compared with its diffractogram after 11 months in shelf conditions (blue), 3 (black), pyrazinamide (green) and AgNO3 (purple), confirming the formation of the new reaction product, 5; Figure S6. 2D fingerprint plots for 1 for the most relevant interactions. Reciprocal contacts are included; Figure S7. 2D fingerprint plots for 2 for the most relevant interactions. Reciprocal contacts are included Figure S8. 2D fingerprint plots for 3 for the most relevant interactions. Reciprocal contacts are included; Figure S9. 2D fingerprint plots for 4 for the most relevant interactions. Reciprocal contacts are included; Figure S10. Percentage of various intermolecular contacts contributing to the Hirshfeld surfaces for 1-4. Reciprocal contacts are included; Figure S11. PXRD patterns of the experimental (red), after 14 months on the shelf at room conditions (blue) and the respective simulated diffractogram from the crystal structure (black) for the synthesised new compounds (a) 1, (b) 2, (c) 3 and (d) 5. For compound 5, the experimental diffractograms (red and blue) are compared with the starting materials: pyrazinamide (black) and silver nitrate (grey), as its structural elucidation was not possible; Table S1. Selected bond lengths (Å) and angles (°) for 1; Table S2. Selected bond lengths (Å) and angles (°) for 2; Table S3. Selected bond lengths (Å) and angles (°) for 3; Table S4. Selected bond lengths (Å) and angles (°) for 4.
