# Supplementary material for: Antimicrobial Activity of Pyrazinamide Coordination Frameworks Synthesized by Mechanochemistry
Source: Molecules. 2021 Mar 28;26(7):1904. doi: 10.3390/molecules26071904 (PMC8036749; doi:10.3390/molecules26071904)
Supplement: Supplementary file 1 [file molecules-26-01904-s001.pdf]

SUPPORTING INFORMATION

Antimicrobial Activity of Pyrazinamide Coordination Frameworks Synthesised by Mechanochemistry

Sílvia Quaresma [1], Paula C. Alves [1,2], Patrícia Rijo [3,4], M. Teresa Duarte [1,5,*] and Vânia André [1,2,*]

[1] Centro de Química Estrutural, Instituto Superior Técnico, Universidade de Lisboa, Av. Rovisco Pais, 1049-001 Lisboa, Portugal
[2] Associação do Instituto Superior Técnico para a Investigação e Desenvolvimento (IST-ID), Av. Rovisco Pais, 1049-003 Lisboa, Portugal
[3] Universidade Lusófona's Research Center for Biosciences and Health Technologies (CBIOS), Campo Grande 376, 1749-024 Lisboa, Portugal
[4] Research Institute for Medicines (iMed. ULisboa), Faculty of Pharmacy, Universidade de Lisboa (UL), Av. Prof. Gama Pinto, 1649-003 Lisboa, Portugal
[5] Departamento de Engenharia Química, Instituto Superior Técnico, Universidade de Lisboa, Av. Rovisco Pais, 1049-001 Lisboa, Portugal

Table S1. Selected bond lengths (Å) and angles (°) for **1**.

Zn(1) - O(1)	2.064(3)	O(1) - Zn(1) - O(2)	172.71(15)
Zn(1) - N(4)	2.193(5)	O(1) - Zn(1) - N(4)	97.09(15)
Zn(1) - O(2)	2.073(3)	O(2) - Zn(1) - N(1)	100.98(15)
Zn(1) - N(5)b	2.179(5)	O(3W) - Zn(1) - N(1)	94.90(2)
Zn(1) - O(3W)	2.042(6)	N(1) - Zn(1) - N(4)	90.03(18)
Zn(1) - N(1)	2.180(5)	O(1) - Zn(1) - O(3W)	97.70(18)
		O(1) - Zn(1) - N(5)b	86.43(15)
		O(2) - Zn(1) - N(4)	76.01(15)
		O(3W) - Zn(1) - N(4)	165.13(19)
		N(1) - Zn(1) - N(5)b	162.62(16)
		O(1) - Zn(1) - N(1)	76.42(15)
		O(2) - Zn(1) - O(3W)	89.28(18)
		O(2) - Zn(1) - N(5)b	95.71(15)
		O(3W) - Zn(1) - N(5)b	90.00(2)
		N(4) - Zn(1) - N(5)b	89.34(18)

Symmetry operations used to generate equivalent atoms:
b = x, 3/2 − y, ½ + z

Table S2. Selected bond lengths (Å) and angles (°) for **2**.

		O(1) - Mn(1) - O(1W)	97.54(5)
		O(1) - Mn(1) - O(5)	147.96(4)
		O(1W) - Mn(1) - O(2W)	169.75(5)
Mn(1) - O(1)	2.1570(11)	O(1W) - Mn(1) - N(1)	92.60(5)
Mn(1) - O(5)	2.7754(18)	O(2W) - Mn(1) - O(5)	89.28(5)
Mn(1) - O(1W)	2.1178(14)	O(3) - Mn(1) - O(5)	47.96(4)
Mn(1) - N(1)	2.3433(12)	O(5) - Mn(1) - N(1)	76.01(5)
Mn(1) - O(2W)	2.1640(13)	O(1) - Mn(1) - O(2W)	92.71(5)
Mn(1) - O(3)	2.3161(13)	O(1) - Mn(1) - N(1)	71.99(4)
		O(1W) - Mn(1) - O(3)	85.27(5)
		O(1W) - Mn(1) - N(4)	93.28(5)
		O(2W) - Mn(1) - N(1)	90.82(5)

O(3) - Mn(1) - N(1)	123.75(5)
O(5) - Mn(1) - N(4)	129.11(4)
O(1) - Mn(1) - O(3)	164.01(4)
O(1) - Mn(1) - N(4)	82.93(4)
O(1W) - Mn(1) - O(5)	82.18(6)
O(2W) - Mn(1) - O(3)	84.80(5)
O(2W) - Mn(1) - N(4)	87.69(5)
O(3) - Mn(1) - N(4)	81.19(4)
N(1) - Mn(1) - N(4)	154.78(4)

Table S3. Selected bond lengths (Å) and angles (°) for **3**.

Ag(1) - N(1)#1	2.230(5)		
Ag(1) - N(1)	2.230(5)	N(1)#1 - Ag(1) - N(1)	154.1(3)
		C(1) - N(1) - Ag(1)	121.3(4)
Symmetry transformations used to generate equivalent atoms: #1 x , −y + 3/2, z		C(4) - N(1) - Ag(1)	122.0(4)

Table S4. Selected bond lengths (Å) and angles (°) for **4**.

Ag(1) - O(2)	2.660(6)	O(2) - Ag(1) - N(1)	119.49(17)
Ag(1) - N(1)	2.227(4)	N(1) - Ag(1) - O(2)a	84.64(17)
Ag(1) - O(2)a	2.660(6)	O(2) - Ag(1) - O(2)a	74.46(18)
Ag(1) - N(1)a	2.227(4)	N(1) - Ag(1) - N(1)a	150.99(18)
		O(2) - Ag(1) - N(1)a	84.64(17)
Symmetry operations used to generate equivalent atoms: a = 1 − x, y, 3/2 − z		O(2)a - Ag(1) - N(1)a	119.49(17)

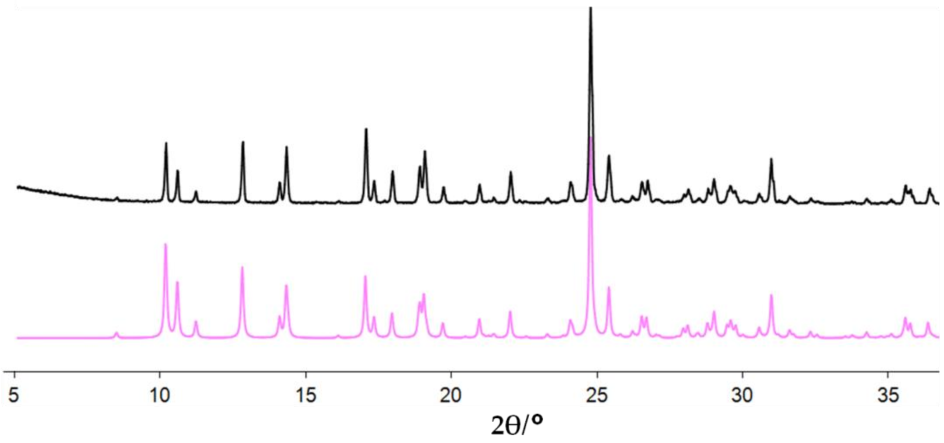

Figure S1. PXRD patterns of the experimental (top) (**1**) and simulated (bottom) of HAYKUP.

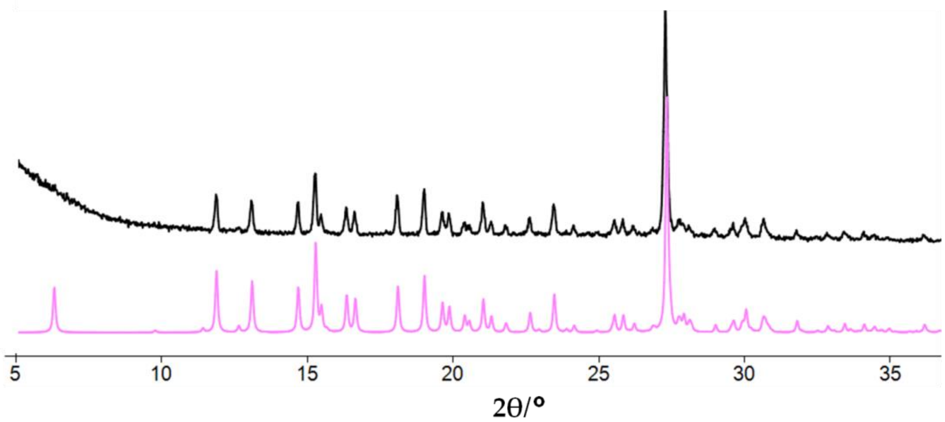

Figure S2. PXRD patterns of the experimental (top) and simulated (bottom) **2**.

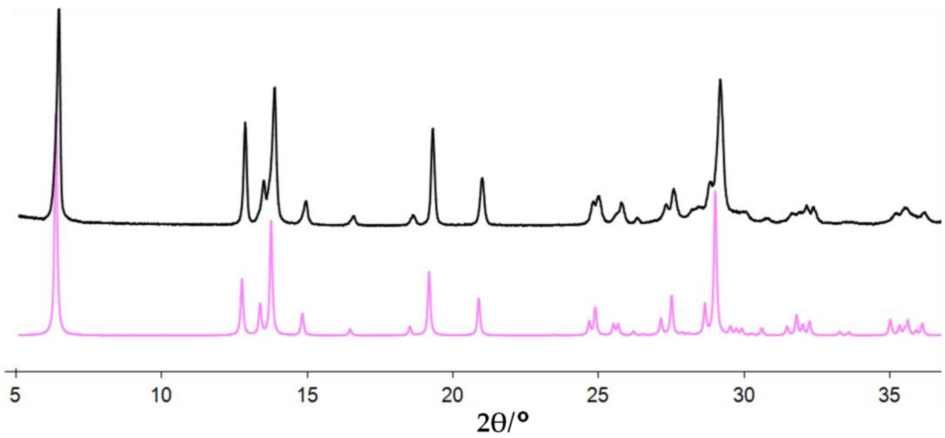

Figure S3. PXRD patterns of the experimental (top) and simulated (bottom) **3**.

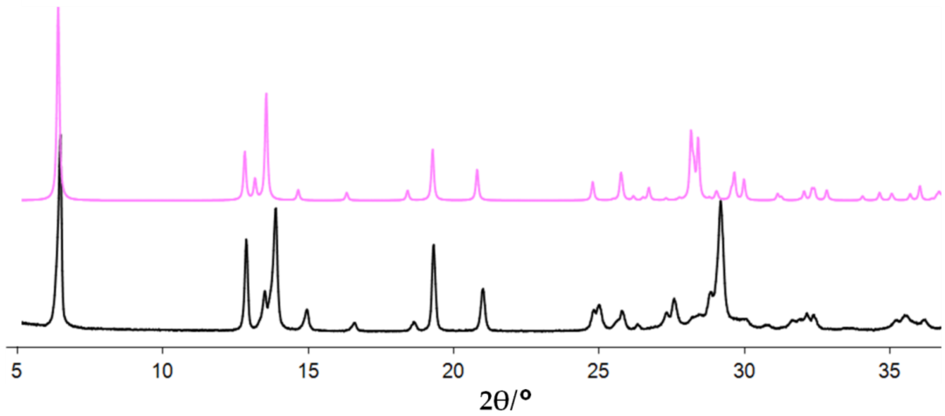

Figure S4. PXRD patterns of the simulated crystal of **4** (top) compared with the experimental **3** (bottom).

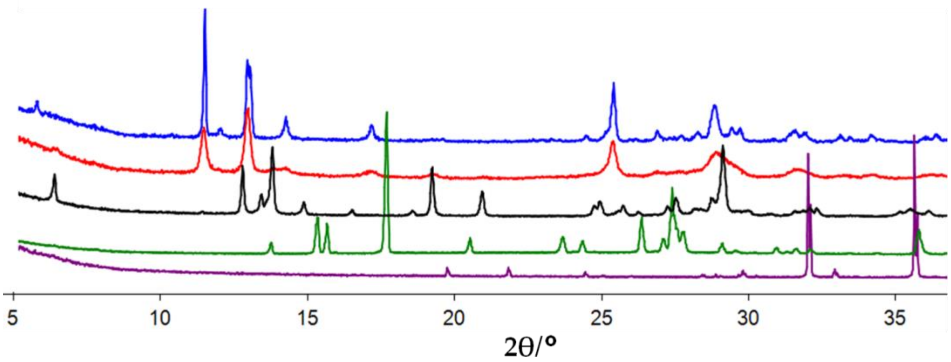

Figure S5. PXRD patterns of the experimental of **5** (red) compared with its diffractogram after 11 months in shelf conditions (blue), **3** (black), pyrazinamide* (green) and $AgNO_3$ (purple), confirming the formation of the new reaction product, **5**. *All pyrazinamide polymorphs were also compared; these comparisons are not displayed for clarity reasons.

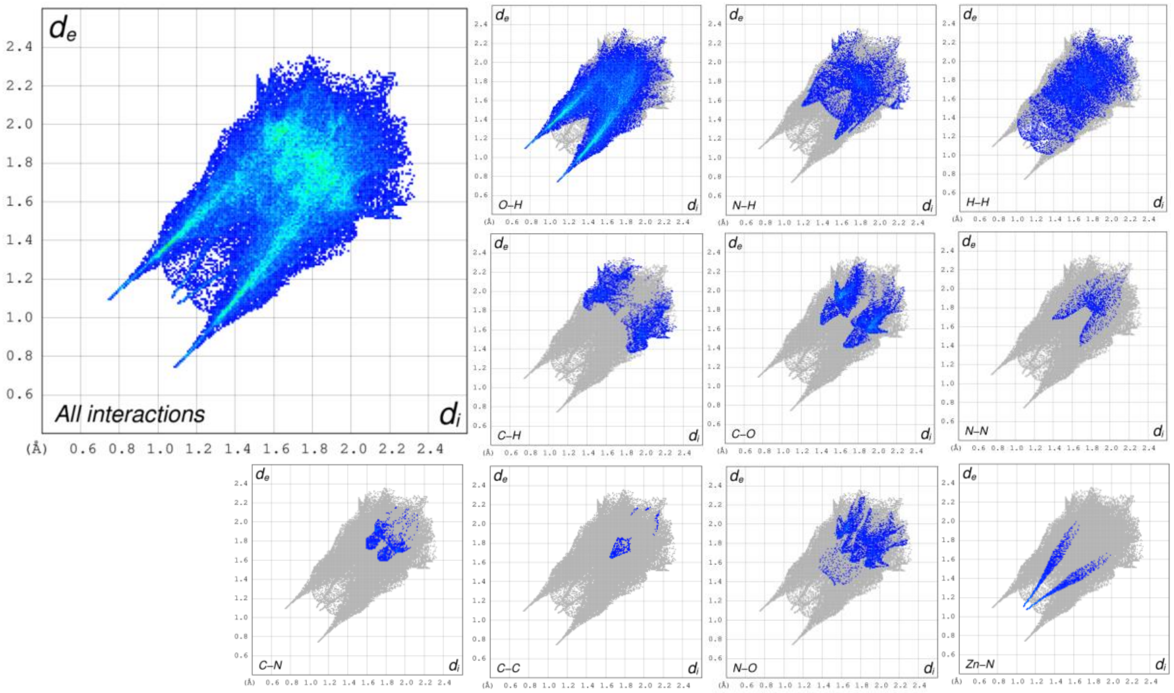

Figure S6. 2D fingerprint plots for **1** for the most relevant interactions. Reciprocal contacts are included.

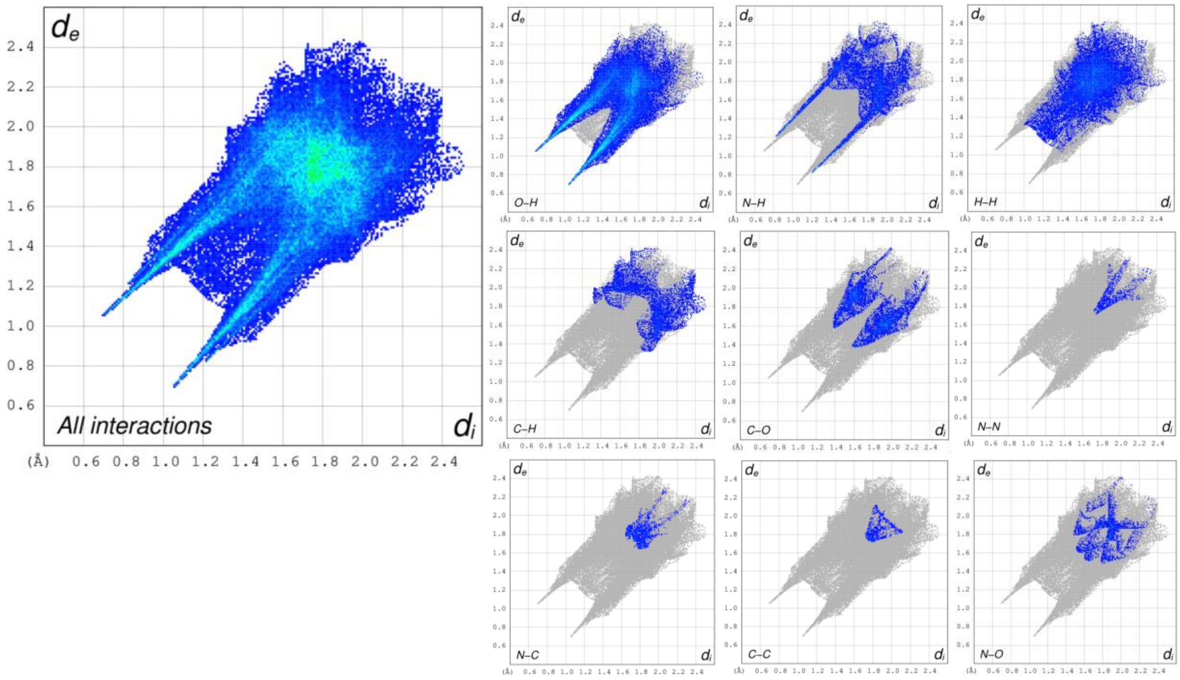

Figure S7. 2D fingerprint plots for **2** for the most relevant interactions. Reciprocal contacts are included.

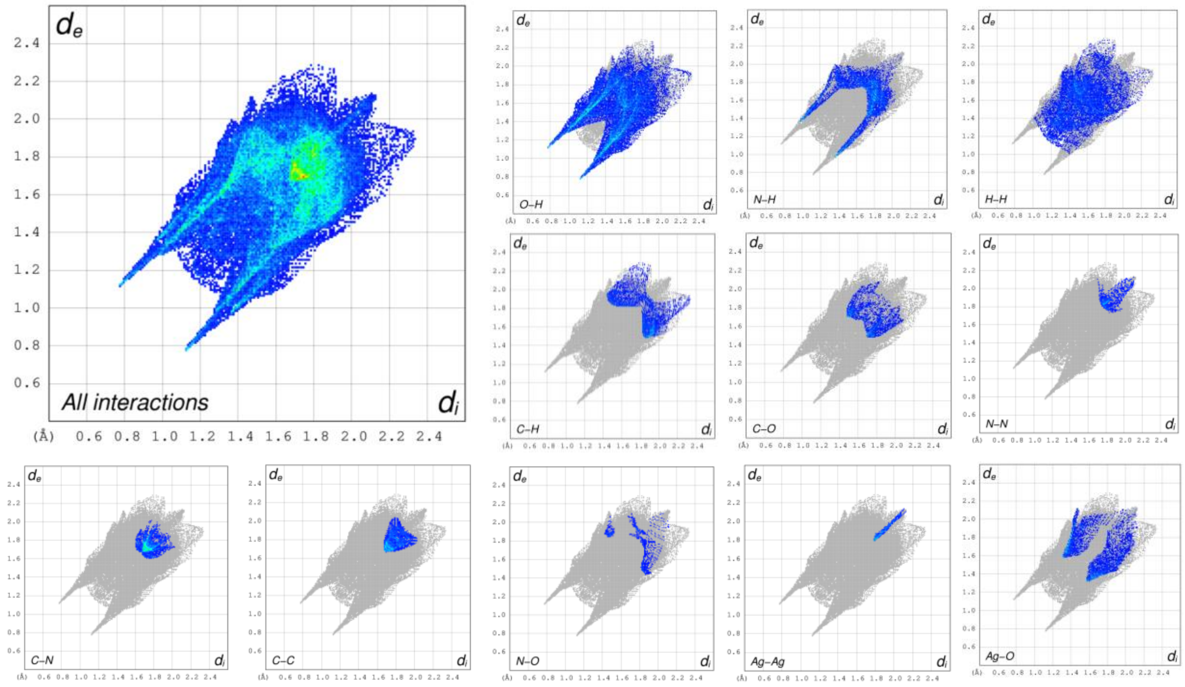

Figure S8. 2D fingerprint plots for **3** for the most relevant interactions. Reciprocal contacts are included.

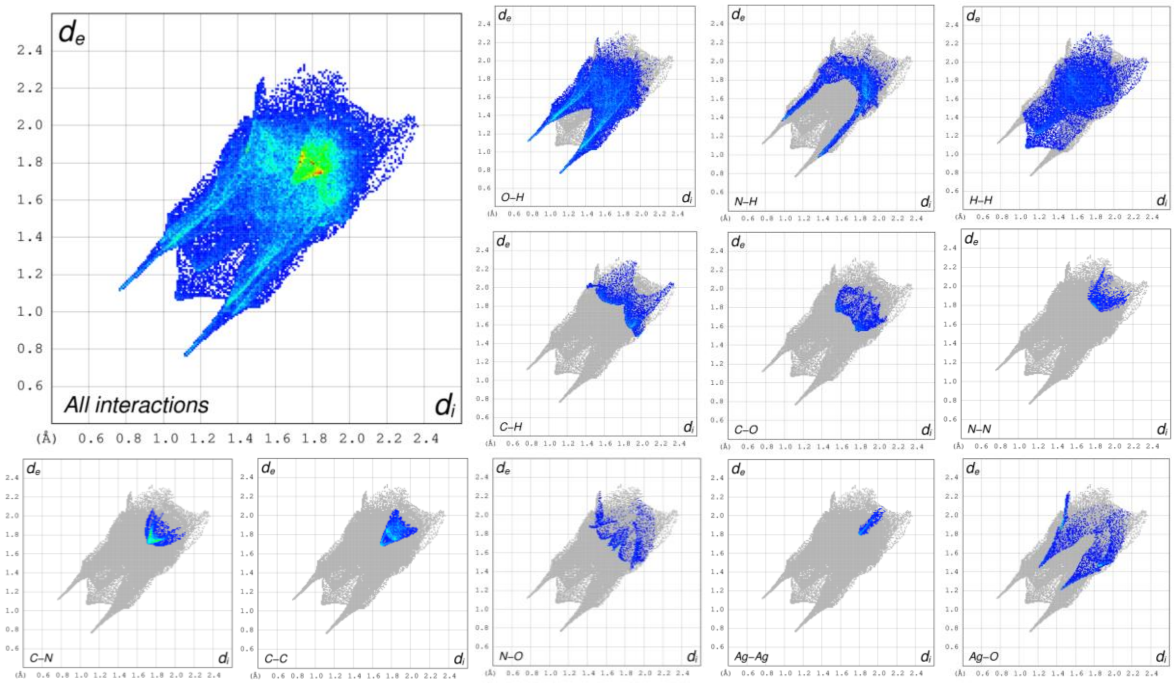

Figure S9. 2D fingerprint plots for **4** for the most relevant interactions. Reciprocal contacts are included.

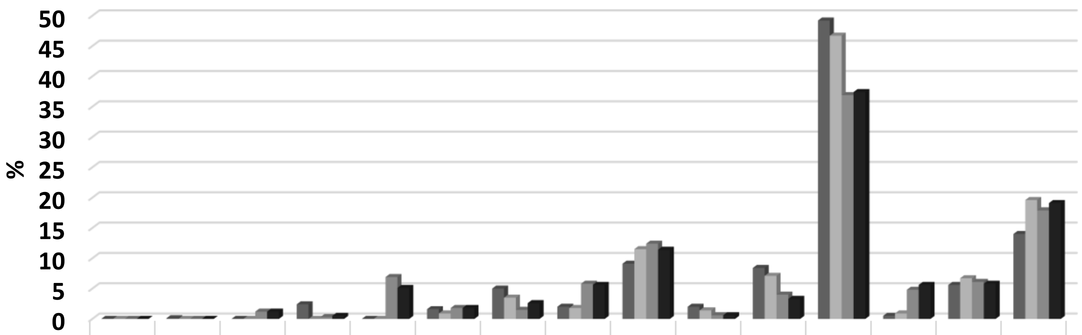

	M-C	M-H	M-M	M-N	M-O	N-N	N-O	N-C	N-H	O-O	O-C	O-H	C-C	C-H	H-H
1	0	0.1	0	2.4	0	1.6	5	2	9.1	2	8.4	49.2	0.5	5.6	14
2	0	0	0	0	0	0.9	3.5	1.8	11.5	1.4	7.1	46.7	0.9	6.7	19.6
3	0	0	1.2	0.3	6.9	1.8	1.5	5.8	12.4	0.6	4	36.9	4.8	6.1	17.9
4	0	0	1.2	0.5	5.1	1.8	2.6	5.6	11.4	0.6	3.3	37.4	5.6	5.8	19.1

Figure S10. Percentage of various intermolecular contacts contributing to the Hirshfeld surfaces for **1–4**. Reciprocal contacts are included.

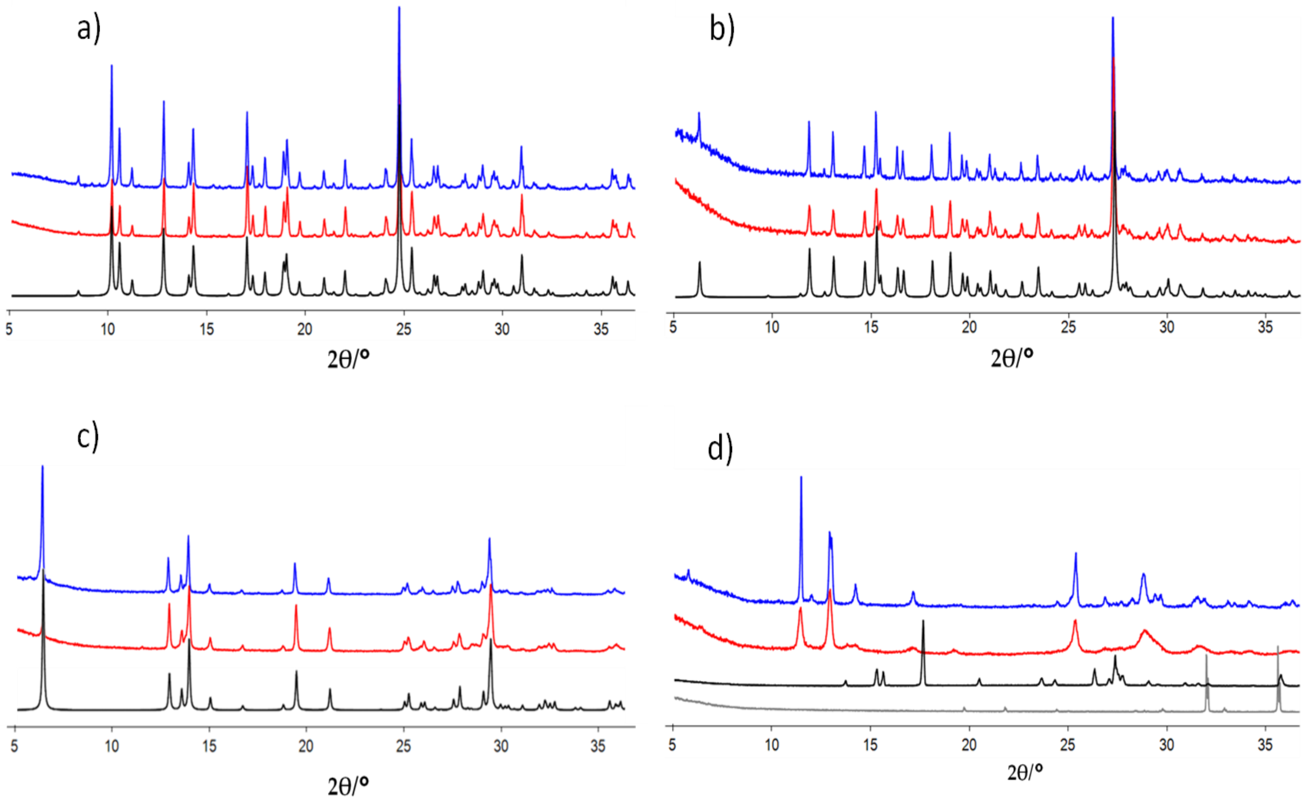

Figure S11. PXRD patterns of the experimental (red), after 14 months on the shelf at room conditions (blue) and the respective simulated diffractogram from the crystal structure (black) for the synthesised new compounds (**a**) 1, (**b**) 2, (**c**) 3 and (**d**) 5. For compound 5, the experimental diffractograms (red and blue) are compared with the starting materials: pyrazinamide (black) and silver nitrate (grey), as its structural elucidation was not possible.
